# The Polymerization of *Aeromonas hydrophila* AH-3 O-Antigen LPS: Concerted Action of WecP and Wzy

**DOI:** 10.1371/journal.pone.0131905

**Published:** 2015-07-10

**Authors:** Susana Merino, Victor Gonzalez, Juan M. Tomás

**Affiliations:** Departamento de Microbiología, Facultad de Biología, Universidad de Barcelona, Diagonal 643, 08071, Barcelona, Spain; University of Helsinki, FINLAND

## Abstract

The repeat units of heteropolymeric O antigen are synthesized at the cytosolic side of the inner bacterial membrane via the Wzx/Wzy-dependent assembly pathway. After being translocated across the membrane by Wzx, each repeat unit is polymerized by Wzy to form a glycan chain. In this study, we demonstrate the need of the corresponding enzyme transferring the initial HexNAc to undecaprenol phosphate (lipid carrier), together with the corresponding O-antigen polymerase (Wzy), to produce the *Aeromonas hydrophila* O:34-antigen. We suggest, the concerted action of WecA or P enzyme (UDP-HexNAc: polyprenol-P HexNAc-1-P transferase) and Wzy is involved in the mechanism responsible for the *A*. *hydrophila* O-antigen polymerization.

## Introduction

Lipopolysaccharide (LPS) is the major component of the outer leaflet of the outer membrane (OM), and is a surface glycoconjugate unique to Gram-negative bacteria. LPS consists of lipid A, core oligosaccharide (OS), and O-specific polysaccharide (O-antigen). O-antigens are glycan chains of homo or heteropolysaccharide repeat units, whose chemical composition, structure, and antigenicity vary widely among Gram-negative bacteria leading to a large number of O-serotypes. [1]. Synthesis of O-antigen subunits starts at the cytosolic face of the inner membrane by the formation of a linkage between the lipid carrier undecaprenyl phosphate (Und-P) and the first sugar 1-phosphate of the corresponding O-antigen unit transferred from a sugar nucleoside diphosphate. An integral membrane protein catalyzes the transfer of glucose (Glc)/galactose (Gal)-1-phosphate (WbaP) or *N*- acetylhexosamine (HexNAc)-1-phosphate (WecA or P) onto Und-P [2, 3]. WecA from *Escherichia coli* is a UDP-HexNAc: polyprenol-P GlcNAc-1-P transferase that transfers GlcNAc to Und-P, while WecP from *Aeromonas hydrophila* is a UDP-HexNAc: polyprenol-P GalNAc-1-P transferase that transfers GalNAc to Und-P [2, 3].

The assembly of the O-antigen after this initial reaction varies depending on the pathways used. Four assembly pathways have been identified, being the Wzx/Wzy- and Wzm/Wzt-dependent schemes the most prevalent while there are few examples of the synthase and Wzk-dependent pathways [4].

In *A*. *hydrophila* the assembly method for heteropolymeric O-antigens follows the Wzx/Wzy-dependent pathway model [5, 6], which is the most widespread O-antigen biosynthesis pathway among bacteria. Following addition of GalNAc-1-P by WecP to Und-P at the cytosolic face of the inner membrane [3], additional glycosyltransferases add two more backbone sugars and a side branch sugar to the undecaprenyl pyrophosphate (UndPP)-linked O repeat. The UndPP-linked O-antigen subunits are then translocated across the membrane by the protein Wzx [7] through a proposed ion-dependent antiport mechanism [8]. Wzx proteins (named flippases) are integral membrane proteins with multiple transmembrane domains [9], and although they carry similar functions they share no similarity in their amino acid residues. On the periplasmic side of the inner membrane, the translocated individual O-antigen subunits are polymerized by the concerted action of Wzy (O-antigen polymerase) [10, 11] and Wzz (O-antigen chain length regulator) [12] to a certain length distribution that is distinct for each O-antigen. In the Wzx/Wzy-dependent pathway the amount of Und-P and WbaP/WecA or P required to build the polymerized O-antigen is several times (depending on the O-antigen repeating units in the final O-antigen) larger than in the Wzm/Wzt pathway, since many O-antigen subunits have to be assembled and translocated across the inner membrane to make the polymerized O-antigen. However, only a single molecule is translocated across the membrane to make the O-antigen in the Wzm/Wzt pathway [13, 14].

Finally, in both pathways an enzyme named WaaL (O-antigen ligase) is able to link the O-antigen completely formed to the lipid A-core OS to produce a complete LPS molecule ready for transport to the outer leaflet of the OM. The WaaL proteins are integral membrane proteins with transmembrane helices and a characteristic large periplasmic loop domain [15, 16].

In the current study, we show that the concerted action of the enzyme mediating the transfer of HexNAc to Und-P (WecA or P) and the O-antigen polymerase (Wzy) is involved in the mechanism responsible for the *A*. *hydrophila* O-antigen polymerization, in the Wzx/Wzy-dependent O-antigen export and assembly pathway.

## Results

**Fig 1:**
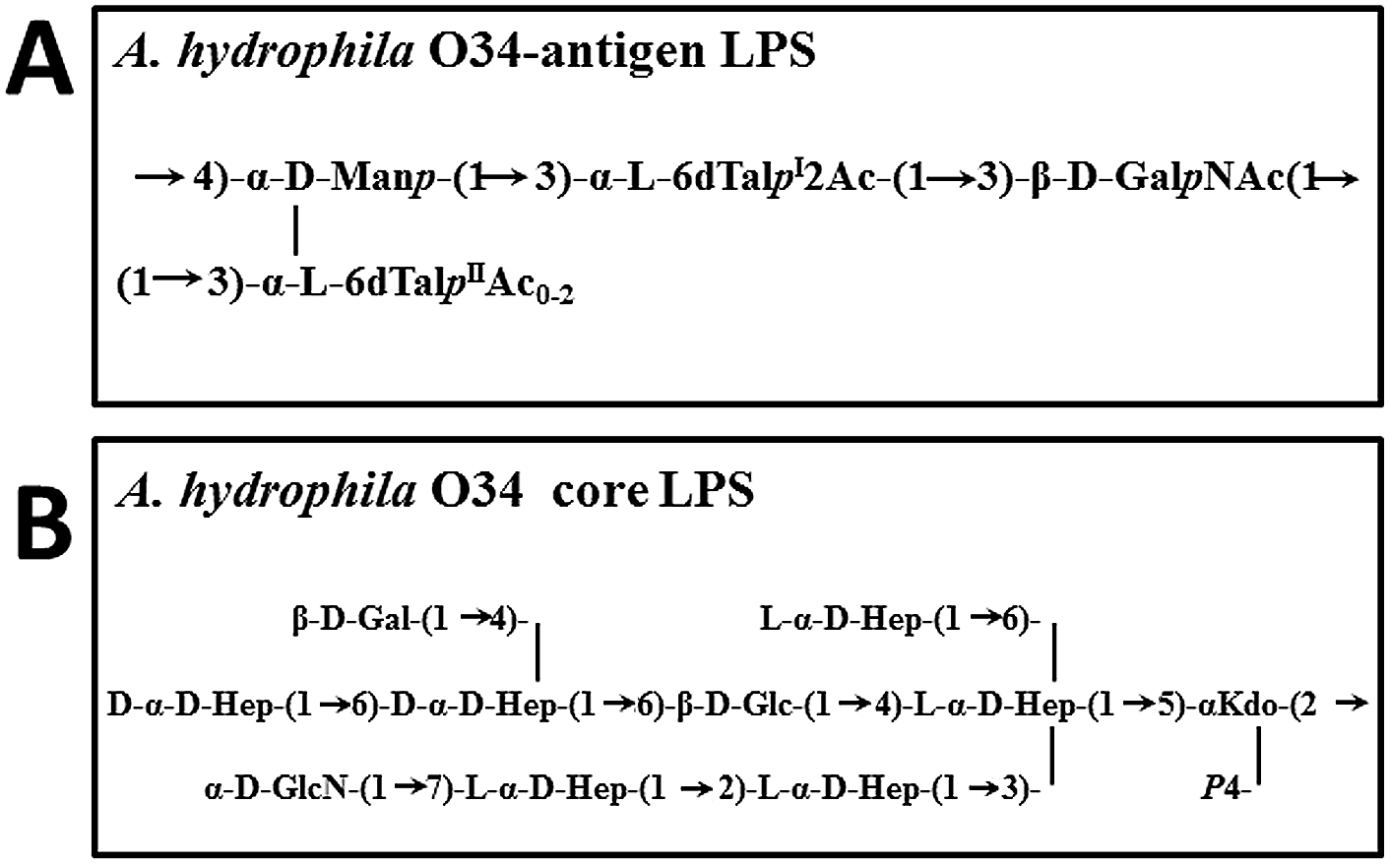
Chemical structure of *Aeromonas hydrophila* LPS. O34-antigen LPS [17] (A) and core LPS [18] (**B**).

The *A*. *hydrophila* O34-antigen repeating subunit is a tetrasaccharide whose proximal sugar is D-GalNAc (Fig 1A) and is linked to the core LPS, previously characterized (Fig 1B) [17, 18]. To obtain this initial sugar *A*. *hydrophila* AH-3 requires the activity of the Gne enzyme which is an UDP-GalNAc4-epimerase responsible for the conversion of UDP-GlcNAc to UDP-GalNAc [19]. Transfer of this sugar to Und-P is performed by WecP which is an UDP-HexNAc:polyprenol-P HexNAc-1-P transferase [3]. In *E*. *coli*, serotypes whose initial O-antigen sugar of the repeating subunit is D-GlcNAc, as in the enterobacterial common antigen (ECA), the transfer of this sugar to Und-P is performed by WecA. After subsequent sugar additions by specific glycosyltransferases, the glycan repeating subunits are exported and assembled by the Wzx/Wzy-dependent pathway. In order to analyze the concerted action of an UDP-HexNAc:polyprenol-P HexNAc-1-P transferase and specific Wzy, we performed cross-complementation studies in different *A*. *hydrophila* mutants.

### Complementation studies on *A*. *hydrophila* AH-3Δ*wecP* mutant

**Fig 2:**
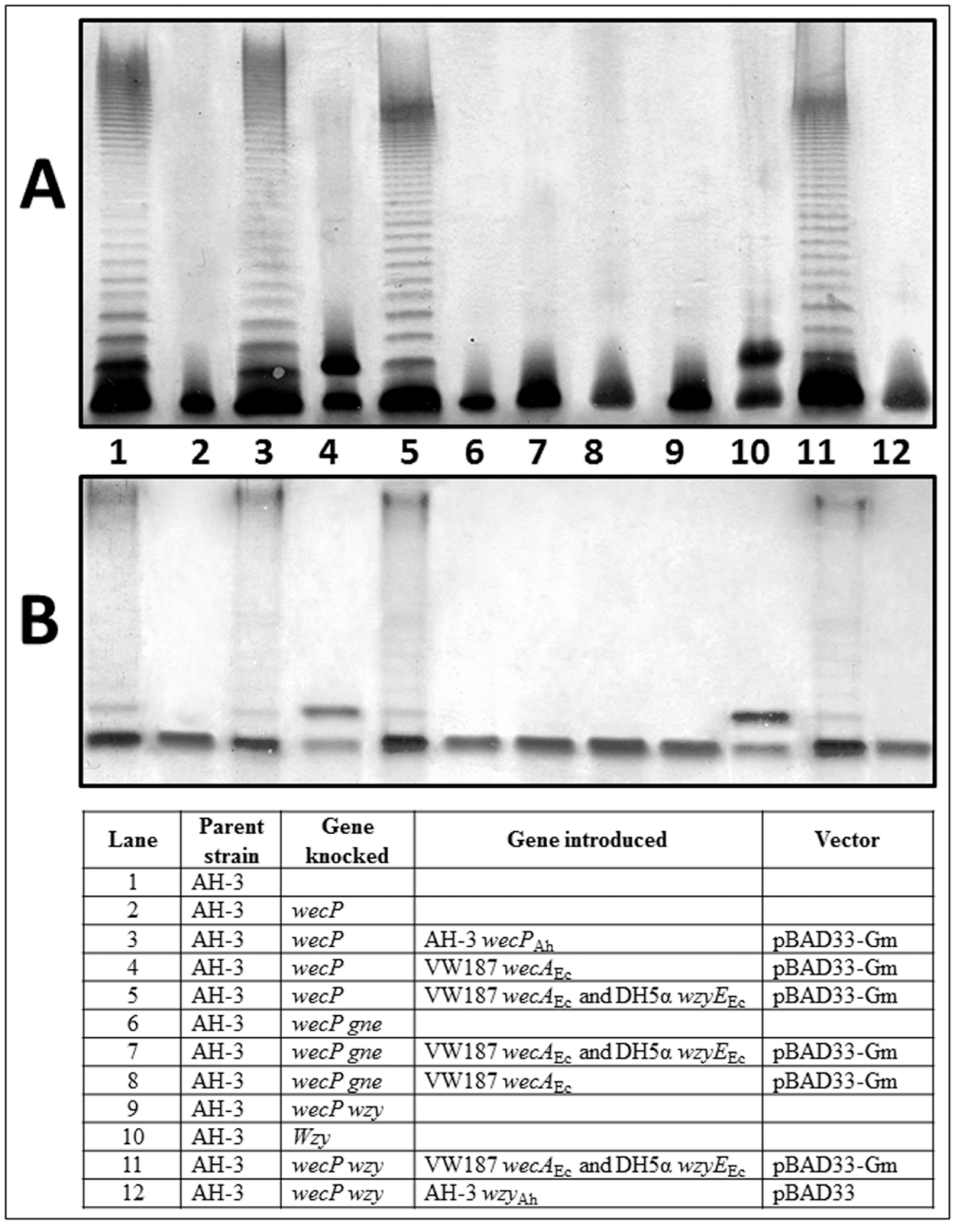
Polyacrylamide gels showing the migration of LPS from AH-3Δ*wecP* mutant and its complementation. The LPS samples were separated on SDS-PAGE (**A**) and SDS-Tricine-PAGE (**B**) and visualized by silver staining. Shown are LPS samples from *A*. *hydrophila* AH-3 (WT) (Lane 1), AH-3Δ*wecP* (Lane 2), AH-3Δ*wecP* + pBAD-WecP_Ah_ (Lane 3), AH-3Δ*wecP* + pBAD-WecA_Ec_ (Lane 4), AH-3Δ*wecP* + pBAD-WecA-WzyE_Ec_ (Lane 5), AH-3Δ*wecP*-*gne* double mutant (Lane 6), AH-3Δ*wecP*-*gne* double mutant + pBAD-WecA-WzyE_Ec_ (Lane 7), AH-3Δ*wecP*-*gne* double mutant + pBAD-WecA_Ec_ (Lane 8), AH-3Δ*wecP*-*wzy* double mutant (Lane 9), AH-3Δ*wzy* double mutant (Lane 10), AH-3Δ*wecP*-*wzy* double mutant + pBAD-WecA-WzyE_Ec_ (Lane 11), and AH-3Δ*wecP*-*wzy* double mutant + pBAD-Wzy_Ah_ (Lane 12) All the strains harbouring pBAD plasmids were grown under induced conditions (+ arabinose) as indicated in Materials and Methods section.

**Fig 3:**
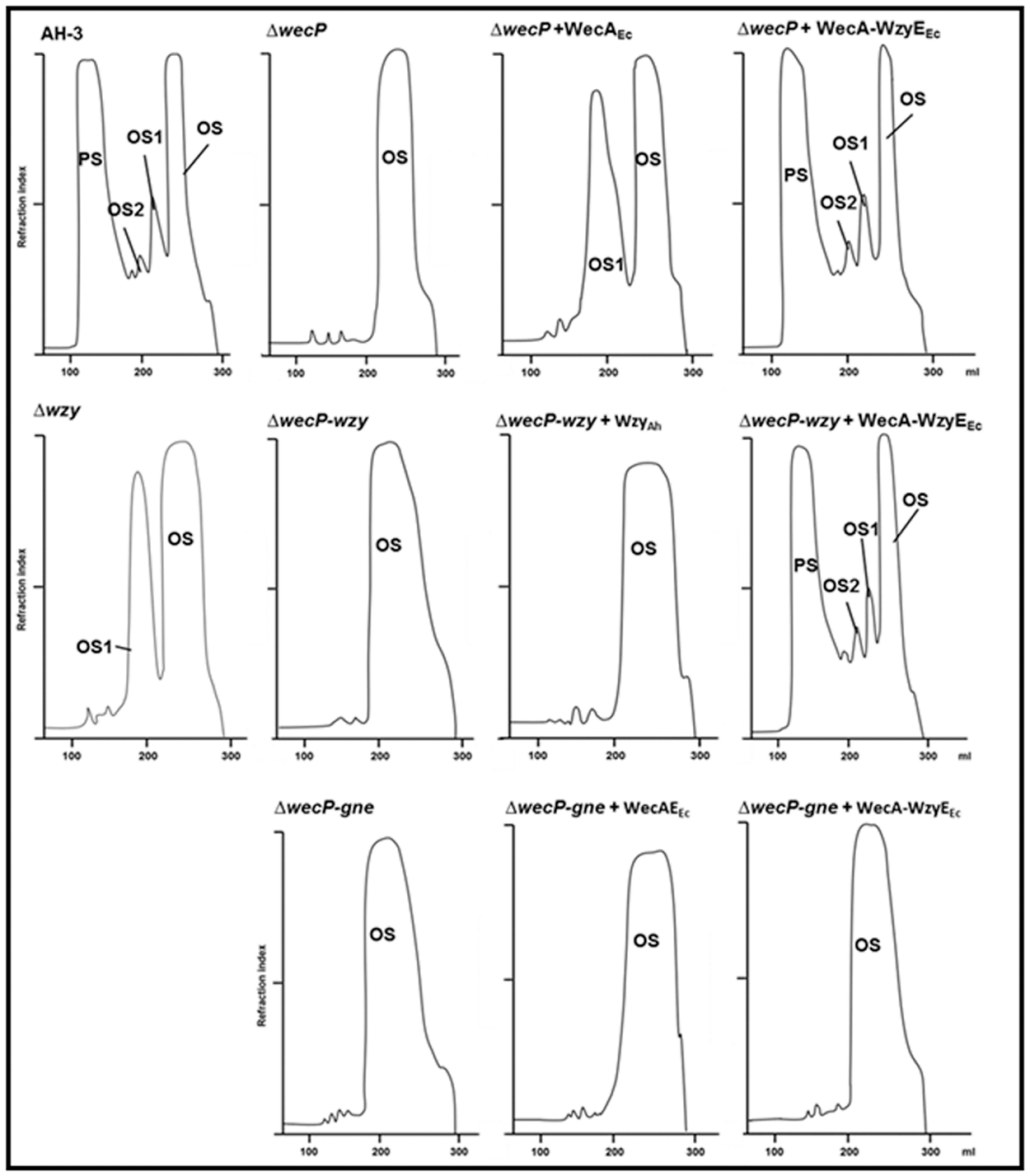
Sephadex G-50 (S) elution profile of the LPS carbohydrate portion. Sephadex G-50 (S) elution profile of the LPS carbohydrate portion from *A*. *hydrophila* AH-3 mutants and its complementation, obtained by mild acid degradation. PS, high-molecular-mass polysaccharide; OS, core oligosaccharide; OS1 and OS2, short-chain polysaccharides containing one and two repeating units attached to the core.

As we previously published, this mutant is unable to add the initial sugar to the Und-P and therefore, is unable to biosynthesize the O34-antigen subunit (Fig 2, lane 2) [3]. The mutant harboring plasmid pBAD33-WecP_Ah_ (carrying the gene from *A*. *hydrophila* AH-3) showed identical LPS banding pattern on gels as their wild type strain (Fig 2, lane 3) while no changes could be observed in the mutant carrying the plasmid vector alone [3]. When plasmid pBAD33-WecA_Ec_ [carrying the *E*. *coli* VW187 (O7) *wecA*] was introduced in the mutant and expressed with arabinose we could see two bands on LPS gels (Fig 2, lane 4; Fig 3) (the band corresponding to core OS and an additional band migrating slowly corresponding to core OS with a single O-unit repeat attached) [3]. Isolated LPS of *A*. *hydrophila* AH-3Δ*wecP* mutant with plasmid pBAD33-WecA_Ec_ grown under expressing conditions (+ arabinose) was devoid of high-molecular-mass O-antigen polysaccharide (O-antigen PS). The sugar analysis of LPS from *A*. *hydrophila* AH-3Δ*wecP* mutant with plasmid pBAD33-WecA_Ec_ grown under expressing conditions (+ arabinose) indicates that LPS molecules showed a single O-antigen repeating unit (Fig 3) [3].

When we introduced plasmid pBAD-WecA-WzyE_Ec_ into *A*. *hydrophila* AH-3Δ*wecP* mutant we could observe a complete complementation when cells were grown under arabinose induction according to their LPS profile banding pattern in gel (Fig 2, lane 5). Neither the plasmid vector alone nor plasmid pBAD33-WecA_Ec_ fully complemented the mutant according to their LPS banding pattern on gels. Isolated LPS of *A*. *hydrophila* AH-3Δ*wecP* mutant with plasmid pBAD-WecA-WzyE_Ec_ grown under inducing conditions (+ arabinose) shows O-antigen LPS in gels according their banding pattern.

**Fig 4:**
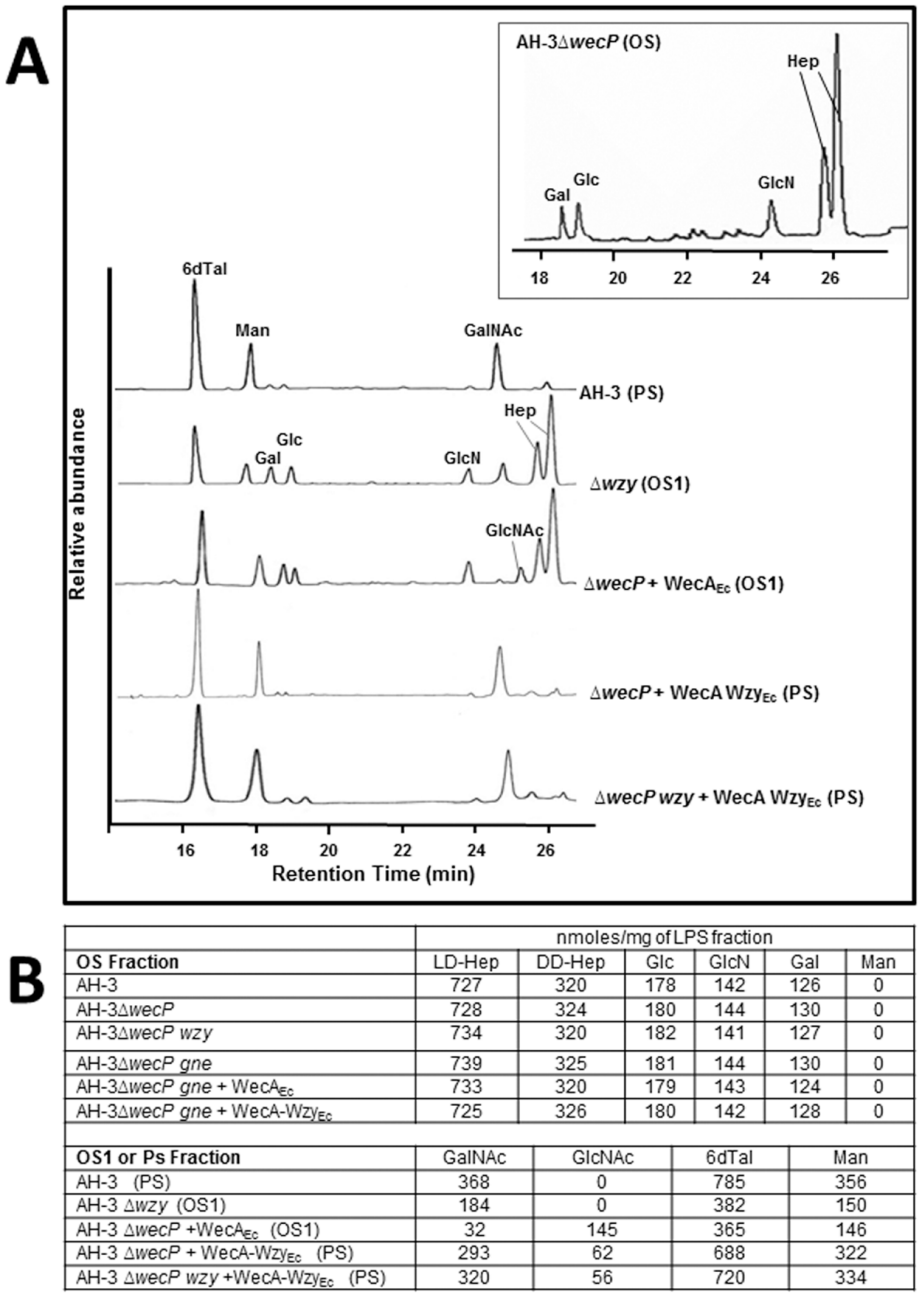
Gas-liquid chromatograms and monosaccharide contents. A) Gas-liquid chromatograms and B) Monosaccharide contents of the LPS fractions from *A*. *hydrophila* AH-3Δ*wecP* mutant and its complementation, determined by GLC. The identity of the polysaccharides is as follow: 6dTal, 6-deoxy-L-talose; Man, D-Mannose; Gal, D-galactose; Glc, D-glucose; GlcNAc, N-acetyl-D-glucosamine; GalNAc, N-acetyl-D-galactosamine; Hep, L-D-heptoses and D-D-heptoses.

The LPS was isolated from enzymatically digested cells of *A*. *hydrophila* AH-3Δ*wecP* + pBAD-WecA-WzyE_Ec_ grown under arabinose inducing conditions (+ arabinose) by phenol/water extraction and purified by ultracentrifugation as indicated in Materials and Methods. After mild acid degradation and GPC fractionation on Sephadex G-50, the high-molecular-mass polysaccharide (PS) was obtained (Fig 3). Sugar analysis of the PS revealed the presence of 6-deoxy-L-talose (L-6dTal) and D-mannose in the ratios 2.1:1, as well as N-acetyl-2-amino-2-deoxy-D-galactose) which are characteristic of *A*. *hydrophila* O34-antigen LPS (Fig 4) [5].

**Fig 5:**
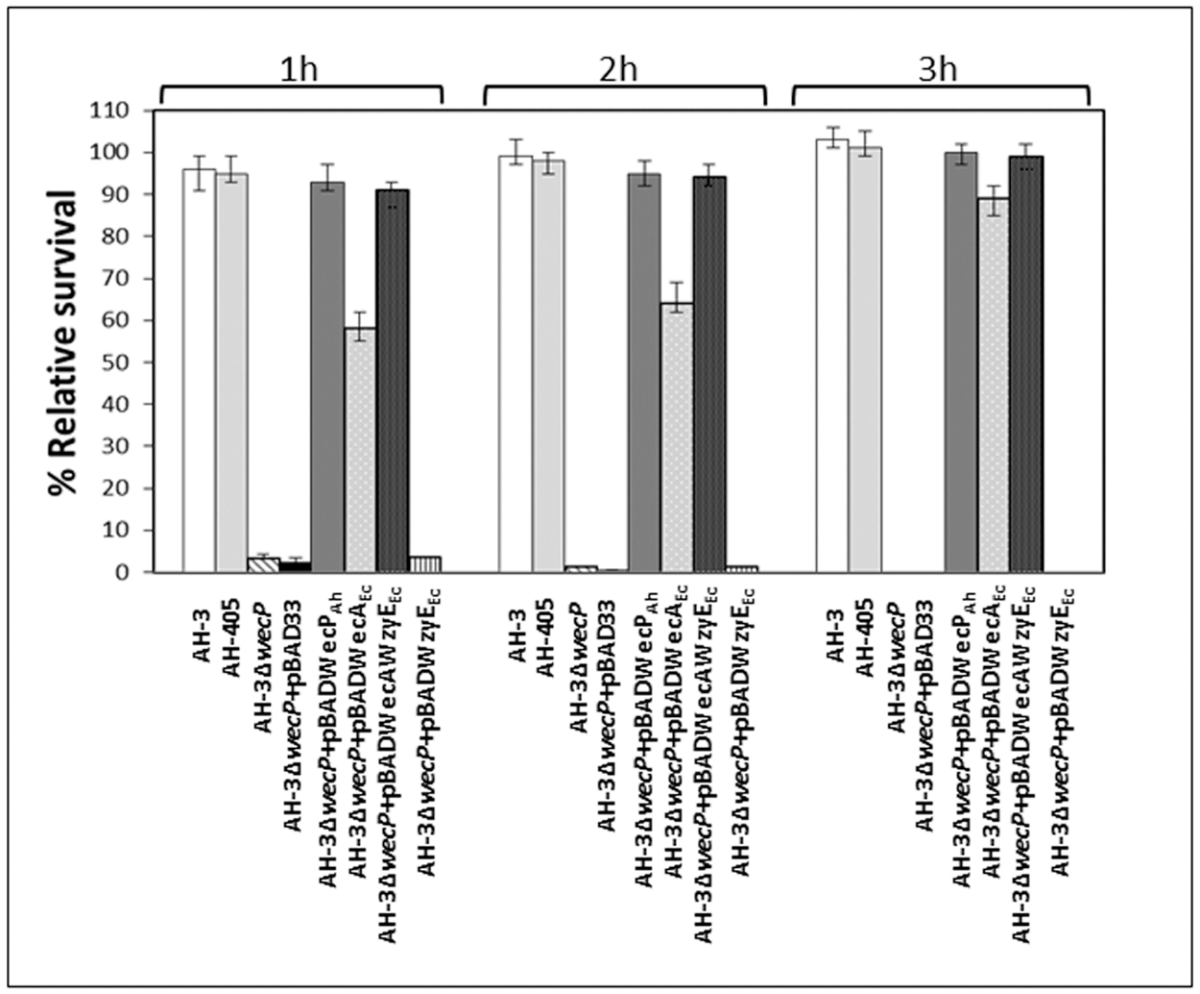
Survival of *A*. *hydrophila* strains in non-immune human serum (NHS). The strains carrying pBAD plasmids were grown under inducing conditions (+ arabinose)

*A*. *hydrophila* whole cells of AH-3Δ*wecP* + pBAD-WecP_Ah_ or pBAD-WecA-WzyE_Ec_ grown under inducing conditions (+ arabinose) were able to positively react in ELISA assays against *A*. *hydrophila* O34-specific antiserum [19]. *A*. *hydrophila* whole cells of AH-3Δ*wecP* + pBAD-WecA_Ec_ grown under inducing conditions (+ arabinose) also showed a positive reaction, though less strong (Table 1). Positive and negative controls were whole cells of *A*. *hydrophila* wild type strain (AH-3) and AH-3Δ*wecP* mutant strain, respectively. We previously showed that the presence of O34-antigen LPS is an important factor for serum resistance [19]. The *A*. *hydrophila* AH-3Δ*wecP* cells were sensitive to nonimmune human serum, while *A*. *hydrophila* AH-3 and AH-3Δ*wecP* + pBAD-WecP_Ah_, pBAD-WecA-WzyE_Ec_ or pBAD-WecA_Ec_ cells grown under inducing conditions (+ arabinose) were resistant (Fig 5). Nevertheless, the survival of AH-3Δ*wecP* + pBAD-WecA_Ec_ is lower in the first times of the process. Table 2 shows that *A*. *hydrophila* AH-3Δ*wecP* cells are less able to adhere to HEp-2 eukaryotic cells than *A*. *hydrophila* AH-3 and AH-3Δ*wecP* with pBAD-WecP_Ah_, pBAD-WecA-WzyE_Ec_ or pBAD-WecA_Ec_ cells grown under arabinose inducing conditions. Several studies indicate that O34-antigen LPS is an adhesion factor to eukaryotic cells [19]. Finally, *A*. *hydrophila* AH-3Δ*wecP* showed a LD_50_ of 10^8.5^ and 10^7.1^ in Swiss mice and Rainbow trout, respectively, while the AH-3Δ*wecP* + pBAD-WecP_Ah_ or pBAD-WecA-WzyE_Ec_ grown under inducing conditions (+ arabinose) showed a LD_50_ ranging between 10^7.5^–10^7.6^ in Swiss mice and 10^5.5^–10^5.6^ in Rainbow trout, which are similar to the wild type strain (10^7.4^ and 10^5.3^) (Table 3). LD_50_ values for *A*. *hydrophila* AH-3Δ*wecP* + pBAD-WecA_Ec_ in Swiss mice and Rainbow trout were nearly a log higher than in the wild type AH-3 (Table 3). All these data indicate that the O34-antigen LPS molecules produced by strain AH-3Δ*wecP* + pBAD-WecA-WzyE_Ec_ grown under inducing conditions (+ arabinose) are able to perform the same biological roles in the assays performed as the one of the wild type strain.

**Table 1:** ELISA assay of different *A*. *hydrophila* whole cells and polyclonal O34-antigen antiserum from *A*. *hydrophila* AH-3, adsorbed with the rough AH-3Δ*waaL* mutant [20].

Strain and main characteristics	A405 (means ±SD)^a^
AH-3	1.9 ± 0.12
AH-405; AH-3 rifampicin-resistant mutant	1.7 ± 0.2
AH-3Δ *wecP*	< 0.2
AH-3Δ *wecP*+ pBAD33	< 0.2
AH-3Δ *wecP* + pBAD-WecP_Ah_	1.7 ± 0.10
AH-3Δ *wecP* + pBAD33-WecA_Ec_	0.7 ± 0.09
AH-3Δ *wecP* + pBAD-WecA-WzyE_Ec_	1.6 ± 0.13
AH-3Δ *wecP* + pBAD-WzyE_Ec_	< 0.2

^*a*^ The values are the averages of three independent experiments.

The strains carrying pBAD plasmids were grown under inducing conditions (+ arabinose)

**Table 2:** Adhesion of different *A*. *hydrophila* serotype O34 strains to HEp-2 cells.

Strain and main characteristics	Mean n°. of bacteria/ HEp-2 cell +/- SD	% Reduction in adhesion^a^
AH-3; wild type	18.3 +/- 2.2	
AH-405; AH-3 rifampicin-resistant mutant	17.9 +/- 2.0	2 ^b^
AH-3Δ *wecP*	7.2 +/- 0.7	61
AH-3Δ *wecP* + pBAD33	6.9 +/- 1.0	62
AH-3Δ *wecP* + pBAD-WecP_Ah_	17.7 +/- 2.1	3
AH-3Δ *wecP* + pBAD33-WecA_Ec_	12.7 +/- 1.4	30
AH-3Δ *wecP* + pBAD-WecA-WzyE_Ec_	18.0 +/- 2.5	1
AH-3Δ *wecP* + pBAD-WzyE_Ec_	6.7 +/- 0.5	63

^*a*^ The level of adhesion of strain AH-3 was used as 100% value.

^*b*^ Student’s *t* test, *P*, 0.001.

The strains carrying pBAD plasmids were grown under inducing conditions (+ arabinose)

**Table 3:** Virulence for rainbow trout and mice of several *A*. *hydrophila* AH-3 (serotype O34).

Strain and main characteristics	LD50^a^ for:	
	Rainbow trout	Swiss mice
AH-3; wild type	10^5.3^	10^7.4^
AH-405; AH-3 rifampicin-resistant mutant	10^5.4^	10^7.6^
AH-3Δ *wecP*	10^7.1^	10^8.5^
AH-3Δ *wecP* + pBAD33	10^7.2^	10^8.2^
AH-3Δ *wecP* + pBAD-WecP_Ah_	10^5.6^	10^7.6^
AH-3Δ *wecP* + pBAD33-WecA_Ec_	10^6.2^	10^8.0^
AH-3Δ *wecP* + pBAD-WecA-WzyE_Ec_	10^5.5^	10^7.5^
AH-3Δ *wecP* + pBAD-WzyE_Ec_	10^7.3^	10^8.6^

^*a*^ The values are the averages of three independent experiments, and the maximum deviation was always < 10^0.3^.

The strains carrying pBAD plasmids were grown under inducing conditions (+ arabinose)

### Complementation studies on *A*. *hydrophila* AH-3Δ*wecP*-*gne* and AH-3Δ*wecP*-*wzy* double mutants

The *gne* gene encodes an UDP-GalNAc 4-epimerase enzyme responsible for the conversion of UDP-GlcNAc to UDP-GalNAc; the *gne* mutant in *A*. *hydrophila* AH-3 lacks the O34-antigen LPS [19]. The *A*. *hydrophila* AH-3Δ*wecP*-*gne* double mutant lacks completely the O34-antigen LPS according to their LPS profile in gel (Fig 2, lane 6). Their purified LPS showed a complete lack of mannose, a characteristic sugar from the O34-antigen LPS not found in the LPS-core [20]. No changes, according to their LPS profile in gel or presence of mannose in their purified LPS, were observed when plasmid pBAD-WecA-WzyE_Ec_ or pBAD33-WecA_Ec_ was introduced in *A*. *hydrophila* AH-3Δ*wecP*-*gne* double mutant cells grown under inducing conditions (+ arabinose) (Fig 2, lane 7 and 8; Figs 3 and 4).

As we previously published, the *A*. *hydrophila* AH-3Δ*wzy* mutant [20] is only able to assemble a single O34-antigen subunit to the core LPS (Fig 2, lane 10; Fig 3). However, the AH-3Δ*wecP*-*wzy* double mutant cells lack completely the O34-antigen LPS according to their LPS profile in gel (Fig 2, lane 9). AH-3Δ*wecP*-*wzy* double mutant cells harboring plasmid pBAD-WecA-WzyE_Ec_ grown under expressing conditions (+ arabinose) were completely able to produce a clear banding pattern corresponding to O34-antigen LPS according to their LPS profile in gel (Fig 2, lane 11; Fig 3) and the ELISA assay performed with polyclonal O34-antigen antiserum (Table 1). Isolated LPS of *A*. *hydrophila* AH-3Δ*wecP*-*wzy* mutant with plasmid pBAD-WecA-WzyE_Ec_ grown under expressing conditions (+ arabinose) shows O-antigen LPS molecules by sugar analyses of their PS with the characteristics sugars mannose, deoxytalose and N-acetyl-amino-deoxygalactose of O34-antigen LPS (Fig 4) [17]. No changes, according to their LPS profile in gel or presence of mannose in their purified LPS, were observed when plasmid pBAD33-Wzy_Ah_ was introduced in *A*. *hydrophila* AH-3Δ*wecP*-*wzy* double mutant cells grown under inducing conditions (+ arabinose) (Fig 2, lane 12; Fig 3).

## Discussion

The O-antigen units are synthesized individually at the cytosolic side of the inner membrane in the Wzx/Wzy-dependent assembly pathway. However, after being translocated across the membrane by the Wzx (O-unit flippase), each unit needs to be polymerized. The Wzy polymerase transfers the growing chain to the non-reducing end of the new O-subunit, forming a glycosidic bond. Then, the concerted action of Wzy (O-antigen polymerase) and Wzz (O-antigen chain length regulator) is responsible for the polymerization of the O-antigen units to a certain length distribution that is unique to each O-antigen [6].

In the *Aeromonas* background we demonstrated that the presence of the *E*. *coli wecA*-*wzyE* (being both from the ECA cluster) are able to elongate the chain with a single O-antigen unit (only with *E*. *coli wecA*) to a complete polymerization of the O-antigen units with the typical length distribution of O34 serotype. The chemical studies and the immunological reactivity with specific antiserum confirm that it is O34-antigen LPS as the wild type strain. Since, *E*. *coli* WecA catalyzes the transfer of the GlcNAc-1.phosphate moiety from UDP-GlcNAc onto the carrier lipid undecaprenyl phosphate, and the initial sugar of O34-antigen subunit of AH-3Δ*wecP* + pBAD33-WecA_Ec_ is GalNAc, this suggests the presence of an epimerase able to convert GlcNAc-P-P-Und to GalNAc-P-P-Und, as described in *E*. *coli* O157 [21]. Furthermore, all the biological data indicates that it is fully active. The experiments performed with AH-3Δ*wecP*-*gne* and AH-3Δ*wecP*-*wzy* double mutants led us to conclude the following points. The lack of *gne* confirms the need for the GalNAc a characteristic monosaccharide of O34 serotype, and the lack of *A*. *hydrophila* AH-3 *wzy* do not interfere with the production of the O34-antigen LPS. At this point, we can indicate that the concerted action of an Und-P transferase and an O-antigen polymerase from the same strain are needed to transfer the growing units from the Und-P to the non-reducing end of the new O-subunit and polymerize them.

As can be observed the Wzx/Wzy O-antigen biosynthesis t pathway is not strain dependent, as it was suggested in some cases, depends on the UDP-HexNAc:polyprenol-P HexNAc-1-P transferase (WecA/P) and their compatibility with the corresponding Wzy (O-antigen polymerase). Then, we suggested that the concerted action of WecA or P (HexNAc-1-phosphate onto Und-P) and Wzy is involved in the mechanism responsible for the O-antigen polymerization, in the Wzx/Wzy-dependent O-antigen export and assembly pathway.

## Materials and Methods

### Bacterial Strains, Plasmids and Growth Conditions

Bacterial strains and plasmids used in this study are shown in Table 4. *Aeromonas* were grown either in tryptic soy broth (TSB) or tryptic soy agar (TSA) and *E*. *coli* Miller lysogeny broth (LB) and LB Miller agar. Spectinomycin (50 μg/ml), tetracycline (20 μg/ml), chloramphenicol (25 μg/ml), gentamicin (20 μg/ml), kanamycin (50 μg/ml), or ampicillin (100 μg/ml) was added to the different media when required.

**Table 4:** Bacterial strains, and plasmids used.

Strain or plasmid	Relevant characteristics	Source or reference
*E*. *coli* strains		
DH5α	F^-^ *end A hsdR17* (rK^-^ mK^+^) *supE44 thi-1 recA1 gyr-A96* _*80lacZ*M15	[34]
XL1-Blue	*recA1 endA1 gyrA96 thi-1 hsdR17 supE44 relA lac* (F^-^ *proAB lacI*q*Z*_M15 Tn*10*)	Stratagene
S17-1	*hsdR pro recA*, RP4-2 in chromosome Km::Tn7 (Tc::Mu)	[5]
BL21(λD3)	F^-^ *ompT hsdS_B_* (r_B_^-^ m_B_^-^) *gal dcm*(λD3)	Novagen
VW187	O7:K1; clinical isolate	M.A. Valvano
*A*. *hydrophila* strains		
AH-3	O34, Wild type	[5]
AH-405	AH-3, spontaneous Rif^R^	[5]
AH-3*wecP* *	AH-3 *wecP* mutant in frame with pDM4	[5]
AH-3*wecP-gne*	AH-2767 (*gne*), *wecP* mutant in frame	This study
AH-3*wecP*-*wzy*	AH-405Δ*wzy*, *wecP* mutant in frame	This study
Plasmids		
pRK2073	Helper plasmid, Spc^R^	[5]
pKO3	Cm^R^ *sacB* temperature sensitive suicide vector	[35]
pKO3Δ*wecA_O150_*	pKO3 with *wecA* in frame deletion	This study
pGEMT-Gne	pGEM-T vector with complete *gne* of AH-3	[19]
pBAD33	arabinose inducible expression vector, Cm^R^	ATCC
pBAD33-Gm	pBAD33 vector with Gm^R^	[36]
pBAD33-WecP_Ah_	pBAD33-Gm with *A*. *hydrophila* AH-3 *wecP*	[3]
pBAD33-WecA_Ec_	pBAD33-Gm with *E*. *coli* VW187 *wecA*	[3]
pBAD-WecP-Wzy_Ah_	pBAD33-WecP_Ah_ with *A*. *hydrophila* AH-3 *wzy*	This study
pBAD-WecA-Wzy_Ec_	pBAD33-WecA_Ec_ with *E*. *coli* DH5α *wzy* ECA	This study
pBAD-Wzy_Ah_	pBAD33 with *A*. *hydrophila* AH-3 *wzy*	This study
pBAD-WzyE_Ec_	pBAD33 with *E*. *coli* DH5α *wzy* ECA	This study

* Formerly named AH-3*wecA*

### General DNA Methods

Standard DNA manipulations were done essentially as described [22] DNA restriction endonucleases, T4 DNA ligase, *E*. *coli* DNA polymerase (Klenow fragment), and alkaline phosphatase were used as recommended by the suppliers.

### DNA Sequencing and Computer Analysis of Sequence Data

Double-stranded DNA sequencing was performed by using the dideoxy-chain termination method [23] with the BigDye Terminator v3.1 cycle sequencing kit (Applied Biosystem). Oligonucleotides used for genomic DNA amplifications and DNA sequencing were purchased from Sigma-Aldrich. The DNA sequence was translated in all six frames, and all open reading frames (ORFs) were inspected. Deduced amino acid sequences were compared with those of DNA translated in all six frames from nonredundant GenBank and EMBL databases by using the BLAST [24] network service at the National Center for Biotechnology Information and the European Biotechnology Information. ClustalW was used for multiple-sequence alignments [25].

### Mutant Construction

AH-3Δ*wecP*-*gne* and AH-3Δ*wecP*-*wzy* double mutants were obtained from AH-2767 [19, *gne* mutant) and AH-405Δ*wzy* [5, *wzy* mutant] by creating a *wecP* in-frame deletion as previously described [3].

### Plasmid constructions and mutant complementation studies

**Fig 6:**
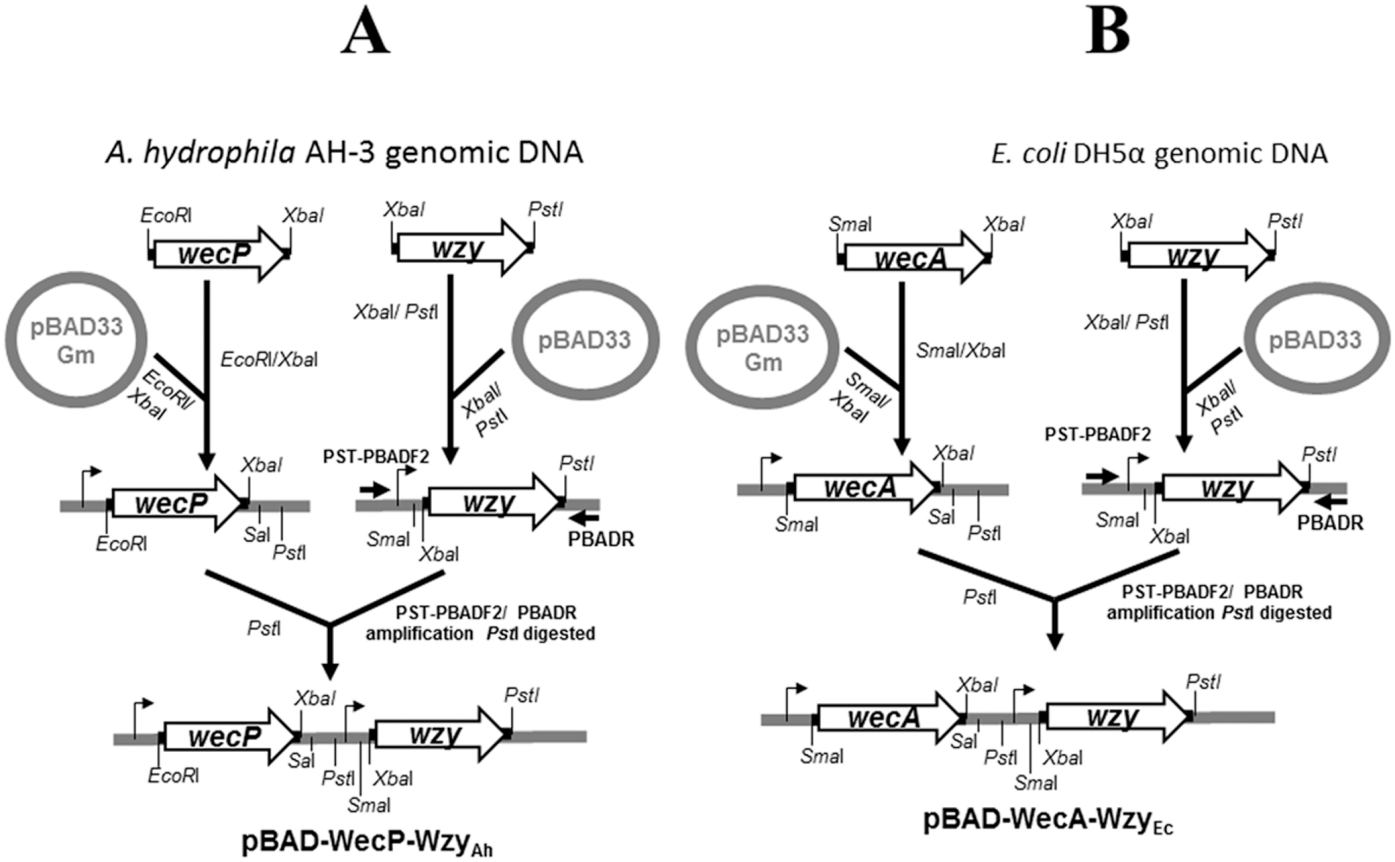
Schematic diagram for plasmids construction. Construction of pBAD-WecP-Wzy_Ah_ from *A*. *hydrophila* AH-3 and described plasmid pBAD33-WecP_Ah_ (A), and pBAD-WecA-Wzy_Ec_ from *E*. *coli* DH5α and described plasmid pBAD33-WecA_Ec_ (B). The detailed methodology is fully described in the Materials and Methods section.

Plasmid pBAD-WecP-Wzy_Ah_ for complementation studies was produced by PCR amplification of the *A*. *hydrophila* AH-3 *wzy* using specific primers pairs 5’-GCTCTAGACACAAGGTTGGTAGTTCC-3’and 5’-AA*CTGCAG*AGGGCAAAAACGCATCAG-3’ to generate a DNA band of 1916 bp, digested with *Xba*I/ *Pst*I and ligated to pBAD33 restricted with same endonucleases. The plasmid with the insert was purified and PCR amplified with oligonucleotides PST-PBADF2/PBADR and the amplified DNA band *Pst*I digested and ligated to pBAD33-WecP_Ah_ [3] digested with the same enzyme to generate pBAD-WecP-Wzy_Ah_ as schematized in Fig 6A. A similar strategy was used to generate pBAD-WecA-Wzy_Ec_. As shown in Fig 6B, PCR amplification of the *E*. *coli wzy* from the ECA cluster using specific primers pairs 5’-GCTCTAGATTGCCGGT GCTGTTTACTA-3’ and 5’-AA*CTGCAG*CGCCAACCAATCAACTGTA-3’ generate a DNA band of 1575 bp, which digested with *Xba*I/ *Pst*I was ligated to pBAD33 restricted with same endonucleases. The plasmid with the insert was purified and PCR amplified with oligonucleotides PST-PBADF2/PBADR and the amplified DNA band *Pst*I digested and ligated to pBAD33-WecA_Ec_ [3] digested with the same enzyme to generate pBAD-WecA-Wzy_Ec_. Oligonucleotides for pBAD33 with the inserts amplification are: PBADR (5’-GGAGACCCCACACTACCAT-3’) and PST-PBADF2 (5’-AAAA*CTGC AG*CGTCACACTTTGCTATGC-3’ with the *Pst*I restriction site underlined).

The plasmid constructions were transformed into *E*. *coli* DH5α by electroporation, plated on gentamycin LB agar plates and incubated at 30°C. Plasmids with the amplified genes were independently transferred into the corresponding mutants by triparental mating using the mobilizing strain HB101/pRK2073 in *Aeromonas* or transformation by electroporation on *E*. *coli*. Transconjugants were selected on plates containing gentamycin (and rifampicin for *Aeromonas*) and confirmed by PCR. Each gene was expressed from the arabinose-inducible and glucose-repressible pBAD33 promoter (P_BAD_). Repression from the *araC* promoter was achieved by growth in medium containing 0.2% (w/v) D-glucose, and induction was obtained by adding l-arabinose to a final concentration of 0.2% (w/v) [26].

### LPS Isolation and Electrophoresis

Cells were grown in LB, washed with water, and dehydrated by sequential washing with methanol:chloroform (1:1) x 3, ethanol, acetone x 2 and diethyl ether. The LPS was extracted from dehydrated cells after evaporation at room temperature of the last dissolvent. The phenol/chloroform/light petroleum ether method [27] was used for strains producing rough LPS (without O-antigen), while the phenol/water procedure [28] was used for the strains producing the O-antigen domain (smooth LPS). For screening purposes LPS was obtained after proteinase K digestion of whole cells [29]. LPS samples were separated by SDS-PAGE or N-[2-hydroxy-1, 1-bis (hydroxymethyl) ethyl] glycine (Tricine)-SDS-PAGE and visualized by silver staining as previously described [30].

### Preparation of Oligosaccharides

The LPS preparations (20 mg) were hydrolyzed in 1% acetic acid (100°C, 120 min), and the precipitate was removed by centrifugation (8,000 x *g*, 30 min) and lyophilized to give Lipid A. The supernatants were fractionated on a column (56 x 2.6 cm) of Sephadex G-50 (S) in 0.05 M pyridinium acetate buffer, pH 4.5, with monitoring using a differential refractometer to obtain the oligosaccharide fractions: high-molecular-mass polysaccharide (PS), an LPS core-oligosaccharide (OS), and sometimes intermediate fractions.

### Gas chromatography-mass spectrometry (GC-MS) analysis

For sugar analysis, the PS was hydrolysed with 2 M CF_3_CO_2_H for 2 h at 100°C, and the monosaccharides were conventionally converted into methylated alditol acetates and methyl glycoside acetates and analyzed on a Agilent Technologies 5973N MS instrument equipped with a 6850A GC and an RTX-5 capillary column (Restek, 30 m x 0.25 mm i.d., flow rate 1 ml/min, He as carrier gas). Acetylated methyl glycosides analysis was performed with the following temperature program: 150° for 5 min, 150°→250° at 3°C/min, 250° for 10 min. For partially methylated alditol acetates the temperature program was: 90°C for 1 min, 90°C →140°C at 25°C/min, 140°C→200°C at 5°C/ min, 200°C →280°C at 10°C/min, 280°C for 10 min.

### Bacterial survival in human serum

Bacterial cells (10^8^ CFU) in the logarithmic phase were suspended in 90% serum—PBS and incubated at 37°C. Viable counts were made at different times until 3 h by dilution and plating as previously described [31]. A pool of nonimmune human sera (NHS) was obtained from healthy volunteers. Control experiments using heat-decomplemented NHS were also performed [31].

### ELISA

Enzyme-linked immunosorbent assays (ELISA) using whole cells as antigens were performed as previously described by us [32]. Briefly, plates with whole cells (10^5^ CFU in the exponential growth phase) as antigen were incubated with serial dilutions of anti-O34 serum and the developing antibody was a 2% dilution of affinity-purified goat anti-rabbit immunoglobulin G alkaline phosphatase. Finally, *p*-nitrophenyl phosphate at 1 mg/mL in 50 mM carbonate buffer (pH = 9.6) was added and the A_450_ was recorded after incubation for 30 min. Controls were whole cells in absence of anti-O34 serum but treated with the developing antibody and substrate.

Specific O34 serum was obtained from adult New Zealand white rabbits previously injected with purified LPS from *A*. *hydrophila* AH-3 grown at 20°C in Freund complete adjuvant, followed by two successive injections using Freund incomplete adjuvant at two-week intervals. After two weeks, the animals were bled and serum was collected and purified [31]. This serum was rendered specifically anti-0:34 after extensive adsorption of the serum with the rough strain AH-3Δ3.1 (O-antigen-deficient AH-3ΔWaaL mutant) [20].

### Adherence assay to HEp-2 cell

Tissue culture and the adherence assay were performed as previously described [19].

### Virulence for fish and mice

The virulence of the strains grown at 20°C was measured by monitoring their 50% lethal dose (LD_50_) by the method of Reed and Müench, as previously described [33].

#### (i) Fish

Rainbow trout (12 to 20 g) were maintained in 20-liter static tanks at 17 to 18°C. The fish were injected intraperitoneally with 0.05 ml of the test samples (approximately 10^9^ viable cells). Mortality was recorded up to 2 weeks; all the deaths occurred within 2 to 8 days.

#### (ii) Mice

Albino Swiss female mice (5 to 7 weeks old) were injected intraperitoneally with 0.25 ml of the test samples (approximately 5 x 10^9^ viable cells). Mortality was recorded up to 1 week; all the deaths occurred within 2 to 5 days.

All the experiments were carried out by specialized technical support workers from the animal room of the Biology Faculty from the University of Barcelona under the supervision of a veterinarian. The final cause of death for animals was bacterial septicemia. Mortality was considered to be caused by the bacterium only if the inoculated bacterium was recovered from the studied death animals. The animals were monitored twice a day, and sacrifice by CO_2_ atmosphere asphyxiation at the end of the experiment or by the use of humane endpoints. These studies were carried out in strict accordance with the recommendations in the Guide for the Care and Use of Laboratory Animals of the National Institutes of Health. The protocols were approved by the Ethics Committee of the University of Barcelona (Permit Number: 4211 for fish and 4212 for mice). No animals involved in the LD_50_ test died without human intervention.
